# Supplementary material for: Insight from the association between critical thinking and English argumentative writing: catering to English learners’ writing ability
Source: PeerJ. 2023 Nov 20;11:e16435. doi: 10.7717/peerj.16435 (PMC10666613; doi:10.7717/peerj.16435)
Supplement: Supplemental Information 2 [file peerj-11-16435-s002.doc]

**CTDI–CV Questionnaire**

亲爱的同学：

你好！

这是一份关于思辨能力的调查问卷，共2页。该调查仅作为科研用途，我们将对调查资料严格保密，请放心填写。下面是思辨能力在性格上所表现出来的一些特质。他们当中有些特质可能你是非常赞同的，有些特质可能你是非常不赞同的，选项没有对错，根据你自己的情况来判定它们。先仔细看清每一特质，并确信你己经理解了它的含义，然后参看下表。在相应的题号下，按照程度说明，将对应的数字打勾，以表示你对该项目的赞同程度。

衷心感谢你的配合！

Dear students:

This is a questionnaire about speculative ability, with a total of 2 pages. This survey is only for scientific research purposes, we will keep the survey data strictly confidential, please feel free to fill in. The following are some of the traits that speculative ability manifests in character. You may agree with some of them, and you may disagree with some of them. There is no right or wrong option, and you can judge them according to your own situation. Take a close look at each trait and make sure you understand what it means, then refer to the table below. Under the corresponding question number, according to the degree description, tick the corresponding number to indicate your degree of agreement with the item.

Sincerely thank you for your cooperation!

姓名： 学号： 学习方向：口文科 口理科

Name Number Discipline：口social science 口science

| **Scale：** | **strongly disagree** | **disagree** | **somewhat disagree** | **somewhat agree** | **agree** | **strongly agree** |
| --- | --- | --- | --- | --- | --- | --- |

**Each of the following questions has a row indicating the level and is divided into six scales of grids, please mark you choose with "√" in the box:**

| **items** | **Strongly disagree Strongly agree** | | | | | |
| --- | --- | --- | --- | --- | --- | --- |
| 1. **我看似有逻辑分析能力，但实际并非如此。**  **I appear to be logical, but I am not.** | **口** | **口** | **口** | **口** | **口** | **口1** |
| 1. **面对难题，坚持不懈不如另谋他途。**   **Faced difficulties, it is better to persevere than to find another way.** | **口** | **口** | **口** | **口** | **口** | **口2** |
| 1. **我目睹了犯罪抢劫，法庭要我出庭作证，我担心会惹出麻烦。**   **If** **I witness a criminal robbery and called to testify in court, I will worry about getting in trouble.** | **口** | **口** | **口** | **口** | **口** | **口3** |
| **4.论证一种观点的最佳依据是你当时的感受。**  **The best basis for arguing for an opinion is how you feel at the time.** | **口** | **口** | **口** | **口** | **口** | **口4** |
| **5.我渴望学习有挑战性的东西。**  **I am eager to learn challenging things.** | **口** | **口** | **口** | **口** | **口** | **口5** |
| **6.即便某个观点我很赞同，但若其论据没有说服力，我还是会很介意。**  **If the arguments are not convincing, even if I agree with a certain point of view, I still will be very concerned.** | **口** | **口** | **口** | **口** | **口** | **口6** |
| **7.我担心自己可能存在着自己没有意识到的偏见。**  **I worry that I may have biases that I'm not aware of.** | **口** | **口** | **口** | **口** | **口** | **口7** |
| **8.我不愿意在多种有争议的观点中作出选择。**  **I don't want to choose between multiple controversial views.** | **口** | **口** | **口** | **口** | **口** | **口8** |
| **9.我认为自己是一个逻辑性强的人。**  **I consider myself a logical person.** | **口** | **口** | **口** | **口** | **口** | **口9** |
| **10.一旦我决定做某事，就不会轻易放弃。**  **Once I decide to do something, I don't give up easily.** | **口** | **口** | **口** | **口** | **口** | **口10** |
| **11.社会上违法违规的事情太多，我们不必为之气愤。**  **There are too many things that violate laws and regulations, so we don't have to be angry about them.** | **口** | **口** | **口** | **口** | **口** | **口11** |
| **12.所谓真理，不外乎个人的看法。**  **The so-called truth is nothing more than personal opinion.** | **口** | **口** | **口** | **口** | **口** | **口12** |
| **13.努力解决复杂问题是一种乐趣。**  **Working hard to solve complex problems is a joy.** | **口** | **口** | **口** | **口** | **口** | **口13** |
| **14. 男性和女性具有同等的逻辑思维能力。**  **Men and women have equal logical thinking skills.** | **口** | **口** | **口** | **口** | **口** | **口14** |
| **15. 对某件事如果多数人赞同，少数人反对，我会支持多数人。**  **When the majority agrees on something and the minority disagrees, I will support the majority.** | **口** | **口** | **口** | **口** | **口** | **口15** |
| **16.我的毛病是，一遇到困难就想放弃。**  **My problem is that I want to give up when I encounter difficulties.** | **口** | **口** | **口** | **口** | **口** | **口16** |
| **17.遇到对别人处置不公时，我感到愤愤不平。**  **I feel indignant when others are treated unfairly.** | **口** | **口** | **口** | **口** | **口** | **口17** |
| **18.我极其喜欢探究事物的本质。**  **I really like to explore the nature of things.** | **口** | **口** | **口** | **口** | **口** | **口18** |
| **19.我常常不由自主地分析别人得论证过程。**  **I often can't help analyzing the process of other people's arguments.** | **口** | **口** | **口** | **口** | **口** | **口19** |
| **20.如果某人的见解明显错误，他便没有权利表达自己的看法。**  **If a person's opinion is clearly wrong, he has no right to express his opinion.** | **口** | **口** | **口** | **口** | **口** | **口20** |
| **21.即使有证据证明我是错的，我仍会坚持自己的想法。**  **Even if there is evidence that I am wrong, I will stick to my ideas.** | **口** | **口** | **口** | **口** | **口** | **口21** |
| **22.面对问题时，因为我能作出客观的分析，所以同伴们会找我做决定。**  **When facing a problem, my peers will come to me to make a decision because I can always make an objective analysis,** | **口** | **口** | **口** | **口** | **口** | **口22** |
| **23.我的决定不易受外界干扰。**  **My decisions are less susceptible to outside interference.** | **口** | **口** | **口** | **口** | **口** | **口23** |
| **24.人们处理问题总是从自己的利益出发。**  **People always deal with problems based on their own interests.** | **口** | **口** | **口** | **口** | **口** | **口24** |
| **25.解决问题的最好方法使从他人那里得到答案。**  **The best way to solve problems is to get answers from other people.** | **口** | **口** | **口** | **口** | **口** | **口25** |
| **26.无论讨论什么话题，我都渴望对它有更多的理解。**  **No matter what topic is discussed, I am eager to gain more understanding of it.** | **口** | **口** | **口** | **口** | **口** | **口26** |
| **27.我喜欢有条理地分析复杂问题。**  **I like to analyze complex problems methodically.** | **口** | **口** | **口** | **口** | **口** | **口27** |
| **28.了解他人对事物的看法，对我来说非常重要。**  **It is very important to me to understand other people's perspectives.** | **口** | **口** | **口** | **口** | **口** | **口28** |
| **29. 我能提出有创造性的解决方案。**  **I can come up with creative solutions.** | **口** | **口** | **口** | **口** | **口** | **口29** |
| **30. 我的很多计划都难以实现。**  **Many of my plans are difficult to achieve.** | **口** | **口** | **口** | **口** | **口** | **口30** |
| **31. 看到别人考试作弊，我不介意。**  **I don't mind seeing other people cheating in exams.** | **口** | **口** | **口** | **口** | **口** | **口31** |
| **32. 事物的本质与其表象是一致的。**  **The essence of things is consistent with their appearance.** | **口** | **口** | **口** | **口** | **口** | **口32** |
| **33. 我尽量多学东西，尽管我不知道何时能派上用场。**  **I try to learn as much as possible, even though I don't know when it will be useful.** | **口** | **口** | **口** | **口** | **口** | **口33** |
| **34. 我力图减少武断，少对事物妄作判断。**  **I try to be less assertive, less judgmental about things.** | **口** | **口** | **口** | **口** | **口** | **口34** |
| **35. 要去寻求许多问题的事实真相让我害怕。**  **It terrifies me to seek the truth about issues.** | **口** | **口** | **口** | **口** | **口** | **口35** |
| **36. 作决定时，别人期待我提出合理的标准。**  **I am expected to set reasonable standards when making decisions.** | **口** | **口** | **口** | **口** | **口** | **口36** |
| **37. 一旦考试成绩不理想，我的学习积极性就倍受打击。**  **Once the test results are not satisfactory, my enthusiasm for learning will be hit.** | **口** | **口** | **口** | **口** | **口** | **口37** |
| **38. 处理问题时，应该尽力做到公正客观、不偏不倚。**  **When dealing with problems, we should try our best to be impartial, objective and unbiased.** | **口** | **口** | **口** | **口** | **口** | **口38** |
| **39. 人们认为我作决定时过于冲动、仓促。**  **People think I'm too impulsive and hasty in making decisions.** | **口** | **口** | **口** | **口** | **口** | **口39** |
| **40. 即使到了60岁，我还想学习新东西。**  **Even at 60, I still want to learn new things.** | **口** | **口** | **口** | **口** | **口** | **口40** |
| **41. 我更喜欢需要分析思考的测试，而不喜欢单凭记忆的考试。**  **I prefer tests that require analytical thinking to memorized tests.** | **口** | **口** | **口** | **口** | **口** | **口41** |
| **42. 我坚持自己的观点，没必要让别人来指指点点。**  **I stand by my opinion and no one has the right to ask me for reasons.** | **口** | **口** | **口** | **口** | **口** | **口42** |
| **43. 实现长期目标，对我来说非常困难。**  **Achieving long-term goals is very difficult for me.** | **口** | **口** | **口** | **口** | **口** | **口43** |
| **44. 对问题的多种解决方法，我不愿意去分析哪个更好。**  **There are many solutions to the problem, and I am not willing to analyze which is better.** | **口** | **口** | **口** | **口** | **口** | **口44** |
| **45. 对不同的世界观持开放态度，并没有人们想得那么重要。**  **Being open to different worldviews is less important than one might think.** | **口** | **口** | **口** | **口** | **口** | **口45** |
| **46. 就大多数事物而言，我们永远不可能了解其本质。**  **As with most things, we can never understand their nature.** | **口** | **口** | **口** | **口** | **口** | **口46** |

This is the end of the questionnaire. Thank you again for your cooperation!

问卷到此结束，再次感谢你的配合！
